# Referral criteria for occupational musculo-skeletal diseases: analysis of 7-year (2012–2018) NODIS data

**DOI:** 10.1186/s12995-025-00481-6

**Published:** 2025-10-08

**Authors:** Ping Hui Chen, Po-Ching Chu, Ching-Chun Huang, Chi-Hsien Chen, Yue Leon Guo, Ta-Chen Su, Pau-Chung Chen

**Affiliations:** 1 National Taiwan University Hospital Hsinchu branch, Hsinchu City, Taiwan; 2 National Taiwan University Hospital, Taipei, Taiwan; 3 National Taiwan University, Taipei City, Taiwan

## Abstract

**Objectives:**

Diagnosing occupational diseases (ODs) needs both primary care clinicians and occupational physicians to make clinical diagnoses and evaluate work-relatedness. Thus, a precise and efficient referral mechanism between them could be crucial for the diagnosis of ODs. ICD codes have once been used as referral criteria, yet novel ODs with multiple etiologies, like musculoskeletal diseases (MSDs), make ICD codes poor referral criteria with low positive predictive values. Thus, the aim of our cross-sectional study is identifying criteria that are associated with work-relatedness and may have better positive predictive values to the existing ICD codes.

**Methods:**

Using data from Network of Occupational Diseases and Injuries Service (NODIS), Taiwan’s ODs surveillance system, during 2012 to 2018, we calculate the odds of cases being recognized as probable according to different demographic factors. A binomial regression model is further used to identify predictors of work-relatedness, and subgroup analysis is then carried out for each MSDs diagnosis.

**Results:**

4651 reported cases of occupational MSDs are included in our study, and 2901 (62.37%) cases are probable cases. Using our binomial regression model, characteristics including tenure, gender, sick leaves, industries and job titles are the predictors of work-relatedness of MSDs, and each MSD is associated with a unique set of predictors, which reflects its occupational etiologies and how occupational physicians evaluate work-relatedness.

**Conclusions:**

Using this method, we could not only identify high-risk characteristics and its diagnostic odd ratio (DOR) for each MSD, but also combine different characteristics into a set of referral criteria and calculate the odds of cases being recognized as probable, which could improve referral mechanisms between occupational medicine and other specialties.

## Introduction

To diagnose occupational diseases, occupational physicians, whose expertise includes work-relatedness evaluations, have to cooperate with primary care clinicians to confirm a patients’ diagnosis before further evaluation. Because most patients usually visit primary care clinicians first for their initial evaluation, cases of occupational diseases are more frequently screened by primary care clinicians, rather than occupational physicians.

In 2010, The Health and Occupation Research (THOR) in the UK developed parallel reporting mechanisms for occupational skin diseases and occupational respiratory diseases, operated by clinicians (dermatologists and pulmonologists) and occupational physicians, respectively. For occupational skin diseases, occupational physicians reported 404 cases to the Occupational Physicians Reporting Activity (OPRA), while dermatologists reported 1789 cases to Occupational Skin Disease Surveillance (EPIDERM). For occupational respiratory diseases, occupational physicians reported 158 cases to OPRA, while pulmonologists reported 1811 cases to Surveillance of Work-related and Occupational Respiratory Disease (SWORD).

Thus, developing referral mechanisms between occupational medicine and other specialties is a key factor in the diagnosis of occupational diseases. During the 1990 s, the US mandated hospitals to report patients whose discharge diagnoses are silicosis (ICD-9 502), asbestosis (ICD-9 500), coal worker pneumoconiosis (ICD-9 501), and other pneumoconiosis (ICD-9 505), because they are likely cases of occupational diseases [1]. Furthermore, to facilitate reporting of work-related asthma, one US study surveyed patients whose discharge diagnoses are respiratory conditions due to chemical fumes and vapors (ICD-9 506), pneumonitis due to solids and liquids (ICD-9 507), and respiratory conditions due to other and unspecified external agents (ICD-9 508) in 1991 [2]. Among these patients, 39% cases coded with 506 are work-related, while only 2% cases coded with 507 are work-related. Thus, the US mandates hospitals to report patients whose discharge diagnoses are coded with 506. A follow-up US study further surveyed patients coded with 506 in 1995 [3]. Among them, patients who are male, white, aged between 17 and 39, coded with 506.2 (upper respiratory inflammation due to fumes/vapors) and 506.9 (unspecified respiratory conditions due to fumes/vapors), are likely cases of occupational diseases with a positive predictive value (PPV) more than 50%.

The aforementioned studies show that, if PPV of disease diagnosis is high enough, it could be used as a referral criterion. Even if its PPV is not high enough, combining disease diagnosis with other characteristics, like gender, age, or other demographic factors could still enhance its PPV. Thus, for occupational diseases with single etiology and clear causal relationship, like pneumoconiosis, silicosis, and asbestosis, diseases diagnosis could serve as good referral criteria. Meanwhile, for occupational diseases with multiple etiologies and a complicated causal relationship, like asthma, combining other characteristics is necessary to enhance its PPV. Nowadays, most cases of occupational diseases are the later ones, including Musculo-skeletal diseases (MSDs).

Since 2010, Spain has established an Occupational Disease Unit (ODU) and took several measures, including patient referrals and retrospective chart reviews, to facilitate diagnosis of occupational diseases [4]. At the ODU, there are occupational physicians responsible of identifying cases of suspected occupational diseases and evaluating their work-relatedness. From 2010 to 2012, 74 suspected cases were identified via patient referrals, and 39% cases were found to be work-related. Meanwhile, 66 suspected cases were identified via retrospective chart reviews, and 7.6% cases were deemed work-related. Apparently, patient referrals are the more preferred measure with a higher PPV. In 2019, another follow-up Spanish study shows that PPV differs significantly among genders, ages, and diseases diagnoses [5]. Patients who are male (PPV 62.5%), aged 55 and more (PPV 63.2%), diagnosed with MSDs (PPV 51.9%), skin diseases (PPV 52.4%), and hearing impairment (PPV 78.6%) are more likely cases of occupational diseases confirmed by the Occupational Disease Unit.

Similarly, Korea has also analyzed claim data from worker compensation insurance to facilitate diagnosis of occupational diseases. One study identified characteristics which increased approval rate of work-relatedness to more than 80% for each MSD [6]. The identified high-risk characteristics include occupation, average duration of employment, and a period of work interruption; cases with these characteristics are regarded as work-related, which allows omission of on-site investigations. Another study identified characteristics predictive of work-relatedness for cerebrovascular diseases [7]. The identified high-risk characteristics include gender, working hours, and other factors related to work burden, except unpredictable work schedules.

Compared with other countries, the incidence rate of occupational diseases in Taiwan is extremely low, which could be explained by under-diagnosis and under-reporting of occupational diseases [8, 9]. Among these occupational diseases, MSDs are the most reported occupational diseases in Network of Occupational Diseases and Injuries Service (NODIS) and also the most compensated ones in worker compensation insurance [8, 9]. In Taiwan, primary care clinicians would refer cases of suspected occupational diseases to occupational physicians, who is responsible of diagnosing occupational diseases. Occupational physicians would evaluate their work-relatedness based on epidemiological literatures, or Taiwan’s official guideline if their diagnosis is included in worker compensation insurance’s list of occupational diseases. To identify more cases of occupational diseases, some hospitals use diagnosis-based referral mechanisms, which are not suitable to reporting MSD. Thus, the aim of our study is to identify high-risk characteristics for each MSD. These characteristics could predict the work-relatedness and serve as useful referral criteria with high positive predictive value. Based on these findings, we could establish better referral mechanisms between occupational medicine and other specialties, which is more precise and more efficient than current diagnosis-based referral mechanisms.

## Methods

### Study population and data sources

In our cross-sectional study, we extract cases of occupational MSDs from the Network of Occupational Diseases and Injuries Service (NODIS), which is the main occupational disease reporting systems in Taiwan from 2008 until now. Occupational physicians would report suspected cases of occupational diseases to NODIS, who usually visit their clinics for claiming benefits from worker compensation insurance. Three occupational physicians appointed by NODIS would determine each case’s work-relatedness and categorize them into probable (> 50% chance), possible (< 50% chance), and non-related (non-qualified cases) based on their consensus.

For each reported case, occupational physicians are required to report many variables, including reported year, ages, length of tenure, gender, job titles, industries, sick leaves records, employment status, disease diagnosis, occupational exposure, temporality, epidemiological evidence, other etiologies, etc. These data are mainly based on comprehensive history taking at clinics, which could also be supported by additional information provided by patients, like proof of worker compensation insurance enrollment, medical charts, attendance records, and work photos/videos. If necessary, we could also arrange worksite visit and workplace environmental monitoring to obtain more information.

Among these variables, age and tenure are numeric scales self-reported by patients. Industries are categorized by 19 categories (A ~ S) in Standard Industrial Classification System, while job titles are categorized by 10 categories (0 ~ 9) in Standard Occupational Classification System. These two systems are national classification systems widely used in Taiwan. For clarity, some job title categories, which are mentioned in our study, are described as follows: “Elementary labourers” are workers doing simple and regular physical labor, which usually needs strength and stamina. “Plant and Machine Operators and Assemblers” are workers operating machines and assembling products. “Craft and related trades workers” are workers using their own skills to manufacture products. “Service and Sales Workers” are workers introducing and selling products directly to customers. “Tecnicians and associate professionals” are assistant professional workers supervising, solving problems and controlling manufacturing.

Disease diagnoses are categorized by ICD-9 (International Classification of Disease and Related Health Problems, 9th Edition) and worker compensation insurance’s List of Occupation Diseases. To avoid misclassification of NODIS data, we reclassify disease diagnosis for all NODIS cases one by one, based on the variables reported by occupational physicians. Gender, sick leaves records and employment status are binary or true-false questions self-reported by patients, while disease diagnosis, occupational exposure, temporality, epidemiological evidence, and other etiologies are variables answered in text by occupational physicians.

### Ethics approval

NODIS routinely collected these non-identifiable data in advance, which is analyzed in our study afterwards. Thus, no ethics approval was obtained for our study.

### Inclusion and exclusion criteria

**Fig. 1:**
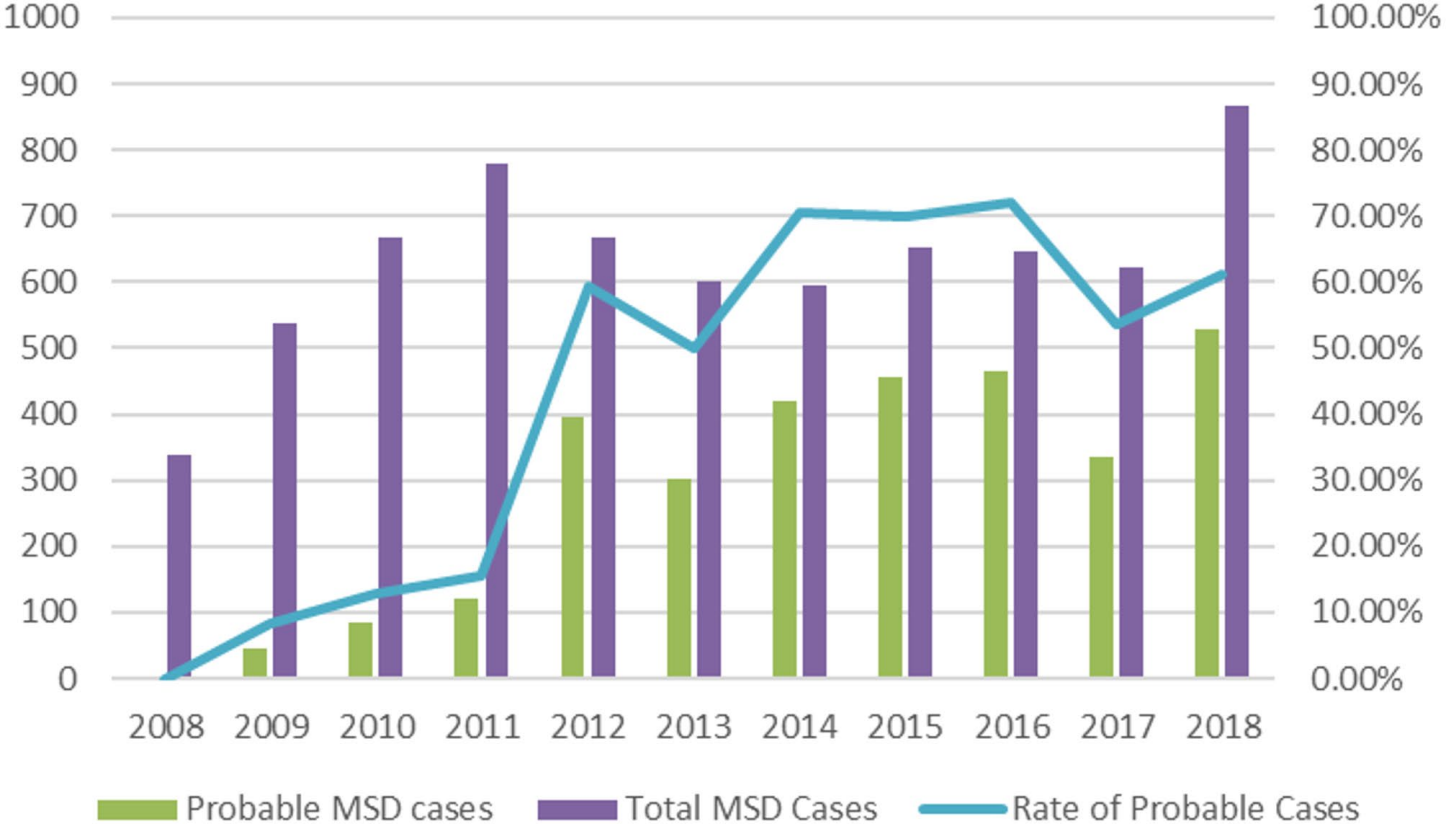
Rate of probable cases of occupational MSDs by years

After examining reported data in NODIS system, we found that work-relatedness evaluation of MSD cases differs significantly between an earlier period (2008–2011) and a later period (2012–2018). As Fig. 1 shows, while less than 20% of reported MSD cases are regarded as probable during earlier period, more than 50% of reported MSD cases are regarded as probable during the later period, when NODIS had more consensus on work-relatedness evaluation. Thus, our study includes only MSD cases during January 1, 2012, to December 31, 2018, which is more representative and could better apply to the current reporting system.

### Statistical analysis

To identify potential high-risk characteristics from numerous reported variables, we first carry out descriptive analysis and present the odds of cases being recognized as probable for each categorical demographic factors, including genders, job titles, industries, sick leaves records, employment status, and disease diagnosis.

Among them, job titles and industries are non-binary characteristics and would be re-grouped based on data distribution for analysis. Categories with less probable cases or lower odds would be combined into others, because they are less likely to be high-risk characteristics. Disease diagnosis is another non-binary characteristic and would be re-grouped by worker compensation insurance’s List of Occupation Diseases. Diagnosis would be divided into listed and non-listed MSDs, and listed MSDs with less probable cases or lower odds would be further combined into minor listed MSDs, including knee osteoarthritis, knee meniscal tear, other upper extremity entrapment neuropathy, cervical spondylosis, etc.

We then use a binomial logistic regression model to investigate whether these characteristics could still remain significant after controlling other variables. In our binomial logistic regression model, the relationship among characteristics and the odds of cases being recognized as probable are as follows:

$$\:f\left(X\right)=\text{ln}\frac{p}{1-p}={{\upbeta\:}}_{0}+{{\upbeta\:}}_{1}\text{*}{\text{X}}_{1}+\cdots\:+{{\upbeta\:}}_{n}\text{*}{\text{X}}_{n}$$

$$\:\gg\:\:\frac{p}{1-p}={e}^{f\left(X\right)}$$

$$\:\gg\:p=\frac{{e}^{f\left(X\right)}}{{1+e}^{f\left(X\right)}}=\frac{1}{{1+e}^{-f\left(X\right)}}\:$$

p: odds of cases being recognized as probable; post-test probability could be calculated if X_1~_ X_n_ are all available.

X: characteristics.

β_n_: coefficient of X_n_, e^βn^ represents Diagnostic Odds Ratio (DOR) of X_n_.

β_0_: constant.

In this binomial logistic regression model, e^βn^ is our main result, which represents Diagnostic Odds Ratio (DOR) [10]. If DOR and its 95% confidence interval (CI) is greater than 1.0, it means this characteristic would significantly enhance p, the odds of cases being recognized as probable. Based on the above Binomial Logistic regression equation, we could even calculate post-test probability, the odds of cases being recognized as probable, if X_1~_ X_n_ are all available [10].

We would perform all the statistical analysis by the software R (version 4.01). The statistical significance level was set at a two-sided p value of < 0.05. 

### Subgroup analysis

We then carry out subgroup analysis to investigate whether each MSD would have its unique characteristics. Among these diagnoses, rotator cuff syndrome (RCS)/bicep tendinitis, carpal tunnel syndrome, lumbar spondylosis, and upper extremity (UE) tendinitis/tenosynovitis are most frequently reported diagnoses. Thus, we carry out the aforementioned descriptive analysis and binomial logistic regression model again to identify high-risk characteristics and its DOR for these four diagnoses, respectively.

## Results

During January 1, 2008, to December 31, 2018, there were 6977 cases of occupational MSDs reported to NODIS. After excluding 2326 cases reported during 2008 to 2011, there were 4651 cases of occupational MSDs included in our study.

Among these 4651 cases, there were 2901 probable cases and 1750 possible cases. Probable cases have higher age (48.88 y/o) and longer tenure (18.72 years) than possible cases (47.85 y/o and 16.41 years). As for categorical demographic factors, as Table 1 shows, the odds of cases being recognized as probable are higher for males, and ever taking sick leaves.

**Table 1:** Odds of cases being recognized as probable and its DOR in binomial logistic regression model by each demographic factors

	Probable cases (%)	DOR (95% CI)
All	2901 (62.37%)	NA
Tenure (per year)	NA	1.01 (1.01–1.02)
Gender		
Female	1327 (59.51%)	1.0
Male	1574 (65.01%)	1.18 (1.03–1.36)
Sick leaves records		
Sick leaves (-)	1808 (60.73%)	1.0
Sick leaves (+)	1093 (65.29%)	1.15 (1.01–1.32)
Employment status		
Keeping jobs	2557 (62.21%)	NA
Losing jobs	344 (63.59%)	NA
Industry categories		
Others	973 (56.47%)	1.0
Construction	651 (70.92%)	1.43 (1.16–1.76)
Manufacturing	839 (64.44%)	1.27 (1.08–1.50)
Accommodation and Food Service Activities	365 (61.45%)	1.31 (1.06–1.60)
Arts, Entertainment and Recreation	73 (64.04%)	1.40 (0.94–2.10)
Job title categories		
Others	477 (53.90%)	1.0
Craft and Related Trades Workers	1007 (67.90%)	1.47 (1.21–1.78)
Technicians and Associate Professionals	410 (64.16%)	1.60 (1.28–1.98)
Elementary Labourers	536 (64.19%)	1.51 (1.23–1.84)
Service and Sales Workers	471 (58.22%)	1.26 (1.03–1.56)
Diseases diagnosis		
Others (non-listed and minor listed MSDs)	122 (37.89%)	1.0
RCS/Bicep tendinitis	471 (62.97%)	2.61 (1.98–3.43)
Carpal tunnel syndrome	970 (64.80%)	3.37 (2.59–4.37)
Lumbar spondylosis	785 (68.14%)	3.34 (2.57–4.35)
UE tendinitis/tenosynovitis	553 (59.33%)	2.71 (2.07–3.55)

“Construction”, “Manufacturing”, “Accommodation and food service activities”, and “Arts, entertainment and recreation” are industry categories with higher odds, while “Craft and related trades workers”, “Technicians and associate professionals”, “Elementary labourers”, and “Service and sales workers” are job title categories with higher odds. As for disease diagnosis, major listed MSDs have higher odds, like RCS/Bicep tendinitis, carpal tunnel syndrome, and lumbar spondylosis. Meanwhile, minor listed MSDs and MSDs not in the list of occupational diseases have lower odds.

Thus, potential high-risk characteristics have been identified, and we put them into a binomial logistic regression model. As Table 1 shows, after adjustment, longer tenures, ever taking sick leaves and male gender are still significant predictors of work-relatedness. “Construction”, “Manufacturing”, and “Accommodation and food service activities” are industry categories with significantly higher DORs, while “Craft and related trades workers”, “Technicians and associate professionals”, “Elementary labourers”, and “Service and sales workers” are also job title categories with significantly higher DORs. As for disease diagnosis, major listed MSDs, including RCS/Bicep tendinitis, carpal tunnel syndrome, lumbar spondylosis, and UE tendinitis/tenosynovitis, are all significant predictors of work-relatedness.

In a subgroup analysis, we then carry out the same descriptive analysis and binomial logistic regression model for each major listed MSD. As Tables 2, 3 and 4 shows, each MSD diagnosis has its unique characteristics with significantly higher DORs, which reflects its occupational etiologies and how occupational physicians evaluate work-relatedness.

**Table 2:** Odds of cases being recognized as probable and its DOR in binomial logistic regression model by each demographic factors for RCS/bicep tendinitis

	Probable cases (%)	DOR (95% CI)
RCS/Bicep tendinitis	**471 (62.97%)**	**NA**
Tenure (per year)	NA	1.03 (1.01–1.04)
Industry categories		
Others	146 (50.69%)	1.0
Construction	169 (73.80%)	1.95 (1.21–3.16)
Manufacturing	106 (63.47%)	1.57 (0.99–2.49)
Other services	36 (75.00%)	2.03 (0.98–4.18)
Electricity and Gas Supply	7 (87.50%)	5.26 (0.63–43.91)
Agriculture, Forestry, Fishing and Animal Husbandry	7 (87.50%)	7.88 (0.94–66.14)
Job title categories		
Others	102 (52.31%)	1.0
Craft and related trades workers	197 (70.36%)	1.45 (0.93–2.26)
Technicians and associate professionals	70 (63.64%)	1.73 (1.02–2.93)
Plant and Machine Operators and Assemblers	30 (66.67%)	1.64 (0.79–3.40)
Service and Sales Workers	72 (61.02%)	1.65 (1.00-2.74)

**Table 3:** Odds of cases being recognized as probable and its DOR in binomial logistic regression model by each demographic factors for carpal tunnel syndrome

	Probable cases (%)	DOR (95% CI)
Carpal tunnel syndrome	**970 (64.80%)**	**NA**
Tenure (per year)	NA	1.01 (1.00-1.02)
Sick leaves records		
Sick leaves (-)	679 (62.01%)	1.0
Sick leaves (+)	291 (72.39%)	1.51 (1.17–1.95)
Industry categories		
Others	155 (53.45%)	1.0
Construction	146 (74.49%)	1.90 (1.22–2.98)
Manufacturing	291 (64.24%)	1.41 (1.03–1.95)
Accommodation and Food Service Activities	202 (68.24%)	2.11 (1.49-3.00)
Wholesale and Retail Trade	64 (70.33%)	2.23 (1.33–3.74)
Other services	80 (60.61%)	1.25 (0.81–1.92)
Agriculture, Forestry, Fishing and Animal Husbandry	19 (86.36%)	6.23 (1.78–21.77)
Education	13 (76.47%)	3.30 (1.04–10.45)
Job title categories		
Others	366 (60.00%)	1.0
Craft and related trades workers	290 (68.24%)	1.40 (1.02–1.91)
Technicians and associate professionals	155 (67.98%)	1.71 (1.20–2.42)
Elementary labourers	159 (67.95%)	1.56 (1.12–2.17)

**Table 4:** Odds of cases being recognized as probable and its DOR in binomial logistic regression model by each demographic factors for lumbar spondylosis and UE tendinitis/tenosynovitis

	Probable cases (%)	DOR (95% CI)
Lumbar spondylosis	**785 (68.14%)**	**NA**
Gender		
Female	124 (58.49%)	1.0
Male	661 (70.32%)	1.50 (1.10–2.07)
Employment status		
Keeping jobs	650 (69.52%)	1.0
Losing jobs	135 (62.21%)	0.70 (0.50–0.96)
Industry categories		
Others	316 (61.84%)	1.0
Construction	236 (75.40%)	1.60 (1.11–2.30)
Manufacturing	233 (71.04%)	1.42 (1.04–1.95)
Job title categories		
Others	456 (60.75%)	1.0
Craft and related trades workers	315 (73.77%)	1.39 (1.00-1.94)
Elementary labourers	193 (71.75%)	1.51 (1.08–2.12)
UE tendinitis/tenosynovitis	**553 (59.33%)**	**NA**
Industries		
Others	243 (54.61%)	1.0
Manufacturing	181 (63.73%)	1.46 (1.08–1.98)
Accommodation and Food Service Activities	105 (61.76%)	1.34 (0.94–1.93)
Arts, Entertainment and Recreation	24 (72.73%)	2.22 (1.01–4.88)

As Table 2 shows, for RCS/Bicep tendinitis, tenure, industry categories, and job title categories are significant predictors of work-relatedness. “Construction” is an industry category with significantly higher DORs, while “Manufacturing”, “Other services”, and “Agriculture, forestry, fishing and animal husbandry” also have higher DORs. “Technicians and associate professionals” is a job title category with significantly higher DORs, while “Service and sales workers”, “Craft and related trades workers”, and “Plant and machine operators and assemblers” also have higher DORs.

As Table 3 shows, for carpal tunnel syndrome, tenure, sick leaves, industry categories, and job title categories are significant predictors of work-relatedness. “Construction”, “Manufacturing”, “Accommodation and food service activities”, “Wholesale and retail trade”, “Agriculture, forestry, fishing and animal husbandry”, and “Education” are industry categories with significantly higher DORs, while “Craft and related trades workers”, “Technicians and associate professionals”, and “Elementary labourers” are also job title categories with significantly higher DORs.

As Table 4 shows, for lumbar spondylosis, male gender, losing jobs, industry categories, and job title categories are significant predictors of work-relatedness. “Construction” and “Manufacturing” are industry categories with significantly higher DORs. “Elementary labourers” is a job title category with significantly higher DORs, while “Craft and related trades workers” also has higher DORs. As for UE tendinitis/tenosynovitis, only industry categories are significant predictors of work-relatedness. “Manufacturing”, and “Arts, entertainment and recreation” are industry categories with significantly higher DORs, while “Accommodation and food service activities” also has higher DORs.

## Discussion

Our study shows that characteristics including tenure, gender, sick leaves, industry categories and job title categories could be predictors of work-relatedness of MSDs. Among these characteristics, some have also been identified by previous studies [5, 6], while others, like sick leaves and job title categories are identified by our study for the first time.

Furthermore, in our subgroup analysis, our study shows that these characteristics differ a lot among different MSD diagnoses, which basically reflects its occupational etiologies and how occupational physicians evaluate work-relatedness. Considering the heterogeneities, it is necessary to analyze and present predictors of work-relatedness for each MSD, rather than all MSDs.

For instance, tenure is an important exposure factor in Taiwan’s guidelines for diagnosis of occupational RCS/Bicep tendinitis (at least 3 months for tendinitis and 12 months for tear or rupture) and Carpal tunnel syndrome (at least 3 months), while the required duration of exposure could be as short as days for occupational UE tendinitis/tenosynovitis. These explain why tenure is a significant predictor only for RCS/Bicep tendinitis and Carpal tunnel syndrome, rather than UE tendinitis/tenosynovitis. Also, male gender and job loss are unique predictors only applied for Lumbar spondylosis, because these are actually part of exposure factors in Taiwan’s guidelines for diagnosis of occupational Lumbar spondylosis. At last, it is also reasonable that high-risk industries and job titles would differ greatly for each MSD, due to its unique occupational etiologies.

For practical applications, we could combine different high-risk characteristics into a set of referral criteria and calculate its post-test probability, the odds of cases being recognized as probable, based on the Binomial Logistic regression equation. Take carpal tunnel syndrome for example. As Table 3 shows, for workers who have ever take sick leaves, and who have worked in Manufacturing industry as Elementary labourers for 10 years, the odds of cases being recognized as probable are

$$\:p=\frac{1}{{1+e}^{-f\left(X\right)}}=\frac{1}{{1+e}^{-(-0.36+0.01\times\:Tenure+0.35\times\:Industry+0.44\times\:\text{J}\text{o}\text{b}\:\text{t}\text{i}\text{t}\text{l}\text{e}\text{s})}}=96.5\%$$

Thus, if we combine these characteristics into a set of referral criteria, it is expected that 96.5% cases would be recognized as probable cases.

Those high-risk characteristics used in the proposed referral criteria, including tenure, gender, industry categories and job title categories, could be easily derived from our worker compensation insurance by data linkage. While other characteristics not recorded by worker compensation insurance, like sick leaves, could still be obtained by primary care physicians with one simple question. Thus, whenever primary care physicians make their diagnosis, combined with these variables, referral mechanism could determine whether patients would be referred to occupational physicians or not, based on the calculated odds of cases being recognized as probable.

This study still has several potential limitations. Firstly, our study group is suspected cases of occupational diseases reported by occupational physicians. These cases are still different from patients referred by clinicians, because occupational physicians already screen patients by their occupational exposure. Thus, to use these high-risk characteristics as referral criteria, we may need additional criteria to screen patients referred by clinicians and exclude cases which are not work-related. However, these additional criteria could not be identified by analysis of NODIS database, which mostly includes suspected cases.

In fact, to identify these additional criteria, we have carried out another small study in our hospital in 2023. Patients aged 20 to 65 years old, diagnosed with MSD included in worker compensation insurance’s list of occupational diseases, and visiting our orthopedics and rehabilitation clinics would be referred to occupational medicine clinics, if they consider their MSD as work-related. During the one-year study period, 24 suspected cases were included into our study. Among them, there were 6 probable cases, 8 possible cases, and 10 non-related cases. All 10 non-related cases have associated occupational exposure of less than 2 h, while 6 of 6 probable cases and 5 of 8 possible cases have associated occupational exposure for more than 2 h. Thus, 2-hour associated occupational exposure could be the additional criteria to screen patients referred by clinicians and exclude cases which are not work-related.

Secondly, NODIS is a database whose accuracy largely depends on reporters. Due to inadequate validation of variables reported by reporters, reporting quality is of concerned, and variables like workplace, job title categories, industry categories, sick leaves records, working status could be misclassified, especially for subgroup analysis, whose case number is relatively small. However, NODIS is the best available and reliable data source in Taiwan. Also, we could still evaluate the magnitude of these misclassifications by checking whether the results of our analysis are reasonable or not, like whether identified characteristics could be explained by guidelines for diagnosis of occupational diseases or its occupational etiologies.

Thirdly, there are still other characteristics, like working hours and other occupational factors, which could also be the predictors of work-relatedness of MSDs. However, these variables are not reported in NODIS database. Thus, identification would only be feasible if NODIS renew its variables or other data source is available.

Thus, our study calls for further intervention studies to establish referral mechanism between occupational medicine and other specialties, and validate whether high-risk characteristics identified by our study could be used as referral criteria, and whether additional criteria would be needed to screen patients referred by clinicians and exclude cases which are not work-related.

## Conclusions

Our study shows that characteristics including tenure, gender, sick leaves, employment status, industry categories and job title categories could be predictors of work-relatedness of MSDs, and each MSD is associated with a unique set of predictors, which reflects its occupational etiologies and how occupational physicians evaluate work-relatedness. Using our Binomial Logistic regression model, we could not only identify high-risk characteristics and its DOR for each MSD, but also combine different high-risk characteristics into a set of referral criteria and calculate the odds of cases being recognized as probable. Thus, based on these high-risk characteristics, we could establish better referral mechanisms between occupational medicine and other specialties, which is more precise and more efficient by having higher odds of cases being recognized as probable.

## Data Availability

Our study’s data source is Network of Occupational Diseases and Injuries Service (NODIS), which is the main occupational diseases reporting system in Taiwan. Data from NODIS is only open to reporters and managers in NODIS and Taiwan’s OSHA.
